# Role of plastoglobules in metabolite repair in the tocopherol redox cycle

**DOI:** 10.3389/fpls.2014.00298

**Published:** 2014-06-26

**Authors:** Lucia Eugeni Piller, Gaétan Glauser, Felix Kessler, Céline Besagni

**Affiliations:** ^1^Laboratoire de Physiologie Végétale, Institute of Biology, Université de NeuchâtelNeuchâtel, Switzerland; ^2^Chemical Analytical Service of the Swiss Plant Science Web, Neuchâtel Platform for Analytical Chemistry, Faculty of Sciences, Université de NeuchâtelNeuchâtel, Switzerland

**Keywords:** Arabidopsis, high light, lipidomics, NAD(P)H dehydrogenase C1, plastoglobule, prenylquinone, redox cycle

## Abstract

Plants are exposed to ever changing light environments and continuously forced to adapt. Excessive light intensity leads to the production of reactive oxygen species that can have deleterious effects on photosystems and thylakoid membranes. To limit damage, plants increase the production of membrane soluble antioxidants such as tocopherols. Here, untargeted lipidomics after high light treatment showed that among hundreds of lipid compounds alpha-tocopherol is the most strongly induced, underscoring its importance as an antioxidant. As part of the antioxidant mechanism, α-tocopherol undergoes a redox cycle involving oxidative opening of the chromanol ring. The only enzyme currently known to participate in the cycle is tocopherol cyclase (VTE1, At4g32770), that re-introduces the chromanol ring of α-tocopherol. By mutant analysis, we identified the NAD(P)H-dependent quinone oxidoreductase (NDC1, At5g08740) as a second enzyme implicated in this cycle. NDC1 presumably acts through the reduction of quinone intermediates preceding cyclization by VTE1. Exposure to high light also triggered far-ranging changes in prenylquinone composition that we dissect herein using null mutants and lines overexpressing the VTE1 and NDC1 enzymes.

## Introduction

In their natural environment, plants are exposed to many kinds of stress, such as heat, drought and high light (Suzuki et al., 2012) especially during summer or due to anthropogenic activities including herbicides, air pollutants and acid rain (Lichtenthaler, 1998). For plants growing under field conditions, occasional exposure to high or even excessive light intensities is normal but has the potential to damage the photosynthetic apparatus. Exposure to high light (HL) generates reactive oxygen species (ROS) in chloroplasts, principally singlet oxygen in PSII (Krieger-Liszkay, 2005) and superoxide in PSI (Asada, 1999). To protect membrane lipids from photooxidation and PSII from photoinhibition, higher plants have developed a variety of adaptive strategies. In addition to modifications of pigment composition (Lichtenthaler et al., 2007) and chloroplast ultrastructure (Austin et al., 2006; Brehelin et al., 2007), plants synthetize various lipid and water soluble antioxidants such as tocopherol (vitamin E) and ascorbate, respectively (Delong and Steffen, 1997; Noctor and Foyer, 1998; Smirnoff, 2000; Smirnoff and Wheeler, 2000; Sattler et al., 2003; Havaux et al., 2005; Van Breusegem et al., 2008). Storage and metabolism of tocopherol but also phylloquinone (Vit K), plastoquinone (PQ) and its derivative plastochromanol (PC8) (Lohmann et al., 2006; Vidi et al., 2006; Szymanska and Kruk, 2010b; Zbierzak et al., 2010; Eugeni Piller et al., 2012) in part take place at chloroplast lipid droplets (plastoglobules; PG) implicating them in light stress responses. PG are attached to the thylakoid membrane by the shared outer lipid leaflet. This arrangement leads to a conduit that may allow the diffusion of lipid molecules between the two compartments (Austin et al., 2006). At least, two metabolic enzymes involved in prenylquinone pathways are located at PG: the tocopherol cyclase VTE1 and NDC1 (Vidi et al., 2006; Ytterberg et al., 2006; Eugeni Piller et al., 2011; Lundquist et al., 2012). In addition, two kinases ABC1K3 and ABC1K1/PGR6 have been implicated in the regulation of PC8 production as well as α-tocopherol overaccumulation under HL. They may function via phosphorylation of VTE1 (Lundquist et al., 2012; Martinis et al., 2013, 2014).

Under HL stress, the synthesis of tocopherol is enhanced suggesting that this molecule exerts an essential role as lipid antioxidant (Munne-Bosch, 2005; Eugeni Piller et al., 2012). It has been shown that tocopherol is important in the maintenance of PSII function (Porfirova et al., 2002; Havaux et al., 2005). A large proportion of total plastid tocopherol is accumulated in the PG core which enlarges during oxidative stress (Vidi et al., 2006; Brehelin et al., 2007).

**Figure 1:**
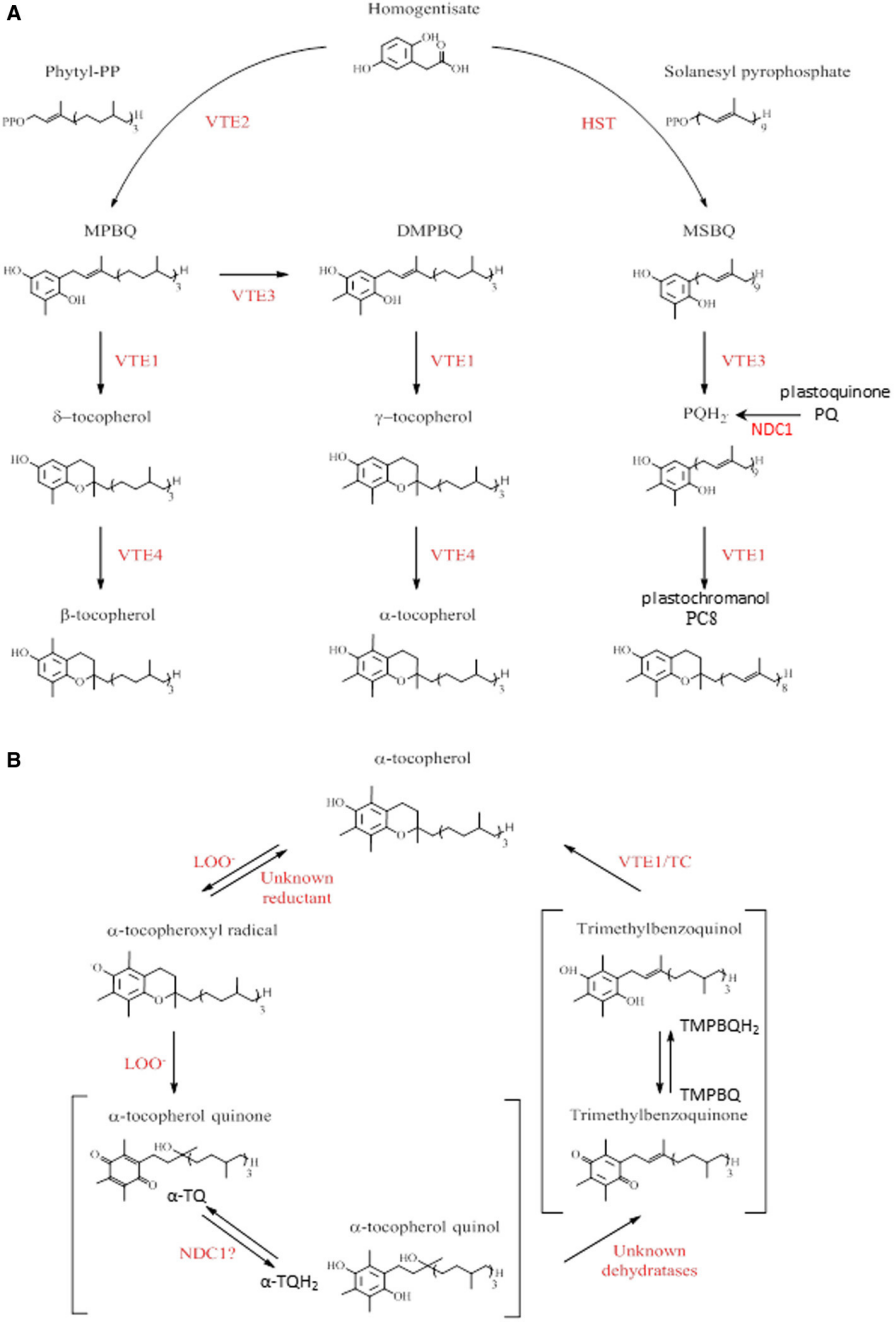
**Tocopherols and tocochromanol biosynthesis and α-tocopherol oxidation pathway in Arabidopsis. (A)** Summary of tocopherols, plastoquinol, and plastochromanol pathways in Arabidopsis. The enzyme abbreviations were shown in red. HST, homogentisic acid solanesyl transferase; VTE, vitamin E synthesis; -PP, pyrophosphate; MPBQ, 2-methyl-6-phytyl-1,4-benzoquinone; DMPBQ, 2,3-dimethyl-6-phytyl-1,4-benzoquinone; MSBQ, 2-methyl-6-solanesyl-1,4-benzoquinol; PQH_2_, plastoquinol; PC8, plastochromanol. Adapted from Dellapenna and Kobayashi (2008), Eugeni Piller et al. (2012). **(B)** α-tocopherol recycling pathway in plants. The enzyme abbreviations were shown in red. LOO-, lipid peroxy radical; TC, tocopherol cyclase. Adapted from Dellapenna and Kobayashi (2008), Mene-Saffrane and Dellapenna (2010), Eugeni Piller et al. (2012).

The tocopherol head group is derived from homogentisic acid that is converted to 2-methyl-6-phytyl-1,4-benzoquinone (MPBQ) by the activity of homogentisate phytyltransferase VTE2 (Figure 1A) (Collakova and Dellapenna, 2001). Then MPBQ is methylated by VTE3 to form 2-3-dimethyl-6-phytyl-1,4-benzoquinone (DMPBQ) (Shintani et al., 2002; Cheng et al., 2003). VTE1 introduces the chromanol ring in MPBQ and DMPBQ leading to δ- and γ-tocopherols, respectively (Porfirova et al., 2002; Sattler et al., 2003). The last methylation step of tocopherol biosynthesis is catalyzed by VTE4 converting the δ- and γ-tocopherol into β- and α-tocopherol, respectively (Shintani and Dellapenna, 1998; Cheng et al., 2003). It has been demonstrated that mutations in Arabidopsis affecting steps of the tocopherol pathway (*vte1* and *vte4* mutants), strongly reduces the tolerance of photosynthetic organisms to HL stress (Maeda et al., 2005; Dellapenna and Pogson, 2006).

In response to HL stress, plants accumulate tocopherol oxidation products. In contrast to animal membranes, only one such product has been reported to accumulate in plants, namely α-tocopherol quinol (α-TQH_2_) (Figure 1B) (Dellapenna and Kobayashi, 2008; Mene-Saffrane and Dellapenna, 2010). A part from being a product of tocopherol oxidation, several functions for α-TQH_2_ have been proposed: dissipation of excess energy, protection of PSII against photoinhibition (Kruk et al., 2000, 2003; Munne-Bosch, 2005) as well as a strong antioxidant activity (Kruk and Trebst, 2008; Nowicka and Kruk, 2010).

Recent studies demonstrate the existence of a plastid-based mechanism for a tocopherol redox cycle (Kobayashi and Dellapenna, 2008; Mene-Saffrane and Dellapenna, 2010; Eugeni Piller et al., 2012). In the first step of this recycling pathway, an α-tocopherol radical is formed by α-tocopherol oxidation via a lipid peroxy radical (LOO^−^). This compound is then oxidized by a second lipid peroxy radical to form α-tocopherol quinone (α-TQ) that is successively reduced to give α-TQH_2_. A yet unidentified plastid-dehydratase activity converts α-TQH_2_ to trimethylbenzoquinone (TMPBQ) (or TMPBQH_2_), which is then cyclized by VTE1 leading to the regeneration of α-tocopherol and completion of the cycle.

In the present study, we used ultra-high pressure liquid chromatography-mass spectrometry to analyse the composition of prenylquinones that play a fundamental role in light stress response in a variety of genetic backgrounds. We report that the PG-localized NAD(P)H-dependent quinone oxidoreductase NDC1 participates in the tocopherol redox cycle. NDC1 most likely functions by reducing α-TQ to α-TQH_2_. This hypothesis is supported by the implication of NDC1 in the analogous reduction of PQ to PQH_2_ in PG under HL stress (Eugeni Piller et al., 2011).

## Materials and methods

### Plant material and growth conditions

*Arabidopsis thaliana* wild-type plants (WT) refers to var Columbia-2 (Col2). In this work, the *ndc1* mutant always corresponds to the T-DNA insertion line SALK_024063 from the Nottingham Arabidopsis Stock Center (http://arabidopsis.info; Alonso et al., 2003). The mutant line *vte1*, obtained by EMS mutagenesis (Porfirova et al., 2002), and the overexpressing 35S:VTE1-YFP plants (Kanwischer et al., 2005) are a gift from Dr. P. Dörmann (Max Planck Institute, Golm, Germany). The 35S:NDC1-YFP plants were obtained as described below.

Plants were grown on soil (Jiffy) under moderate light conditions (150 μmol m^−2^ s^−1^, 22°C, 8/16 h light/dark period) in a controlled environment room. For HL stress, 5 weeks old plants were exposed to 500 μmol m^−2^ s^−1^ (25°C, 8/16 h light/dark period).

### Overexpression of NDC1 in *A. thaliana* leaves

Plants overexpressing NDC1-YFP under the 35S promoter were obtained using the Gateway recombination technology (Invitrogen): The NDC1 coding sequence was introduced into a donor vector pDONR™221, and subsequently transferred into an appropriate destination vector, the pEarlyGate101-YFP binary vector, resulting in pEarlyGate101-NDC1-YFP. pEarlyGate101-NDC1-YFP was transferred into Arabidopsis WT plants using the floral dip method (Clough and Bent, 1998). Transformed plants were selected for BASTA resistance and confirmed by segregation analysis.

### Western blot analysis

Total protein was isolated from Arabidopsis leaves according to Rensink et al. (1998) and concentrated by chloroform-methanol precipitation (Wessel and Flugge, 1984). Twenty μg of protein were separated by SDS-PAGE and blotted onto nitrocellulose membrane for immunodetection. Immunodetection was carried out using anti-NDC1 serum at 1/1000 dilution in 5% fat free milk powder/TBS (Eugeni Piller et al., 2011).

### Confocal microscopy

Protoplasts were released from plants overexpressing NDC1-YFP by overnight digestion with macerozyme (0.25%, Serva) and reduced cellulase (1%, Serva) in a solution containing 400 mM Mannitol, 5 mM MES and 8 mM CaCl_2_. Protoplasts were filtered and loaded on a sucrose gradient (21 and 42%) and centrifuged for 10 min at 50 × g. Intact protoplasts were resuspended and fluorescence was monitored with a Leica TCS SP5 confocal microscope using the appropriate parameters for YFP (514-nm laser lines, 520–588-nm detection windows).

### Prenyllipid extraction from whole plants or PG fractions and lipidomics profiling

Prenylquinones were extracted from whole plants using an established method (Martinis et al., 2011). Leaves were ground in liquid nitrogen in a mortar. 100 mg were re-suspended in 500 μl of tetrahydrofuran (THF, analytical grade, Normapur). Glass beads (1 mm) were added and samples homogenized at 30 Hz, 3 min (Retsch MM 300). After centrifugation, 200 μl were transferred to a suitable HPLC vial.

To measure prenylquinones contained in PG, intact chloroplasts were isolated by centrifugation on a Percoll gradient. Subsequently, PG were separated from thylakoid membranes by flotation on a sucrose gradient as described in Besagni et al. (2011). Four hundred μl of PG and thylakoid fractions were added to 600 μl of water and extracted three times with an equal volume of ethylacetate. Organic phases were pooled, evaporated and pellets were dissolved in 100 μl of THF/water (85/15 v/v) and the solution transferred to an appropriate HPLC vial (Kessler and Glauser, 2014).

The quantification of prenyllipids was performed using reverse-phase ultra-high pressure liquid chromatography (Acquity UPLC™, Waters) coupled to quadrupole-time-of-flight mass spectrometry (Synapt G2, Waters) (UHPLC-QTOFMS). Absolute concentrations of α-tocopherols, α-tocopherol quinone, plastochromanol and plastoquinone were calculated based on calibration curves obtained from pure standards. The method was also used for untargeted lipid profiling (Eugeni Piller et al., 2011; Martinis et al., 2011; Kessler and Glauser, 2014).

### Data pre-processing and statistical analysis

For comparison of metabolic profiles, raw spectrometric data were processed using Markerlynx XS™ (Waters) which performs automatic peak detection and deconvolution in each chromatogram. The parameters were as follows: initial and final retention times 0.5–3.0 min, mass range *m/z* 300–1200 Da, mass tolerance 0.02 Da, retention time window 0.10 min, automatic peak with detection, automatic measurement of peak-to-peak baseline noise, intensity threshold 400 counts, no smoothing, noise elimination level disabled, deisotope filtering function applied. Peak areas for individual variables were normalized to the total integrated area per sample. Variables were Pareto-scaled before applying principal component analysis (PCA).

### Statistical tests

Multivariate statistical analysis of lipid profiles was performed using EZinfo (Umetrics). Univariate Analyses were performed using the software SigmaPlot version 12.0. Data were first analyzed using Shapiro-Wilk to determine whether data were normally distributed. When data passed the test, the Student’s test (*t*-test) was applied to evaluate statistically significant difference between values (*p* < 0.05). For non-normally distributed data, the Mann-Whitney *U*-test was used.

## Results

### Characterization of NDC1 overexpressing plants

**Figure 2:**
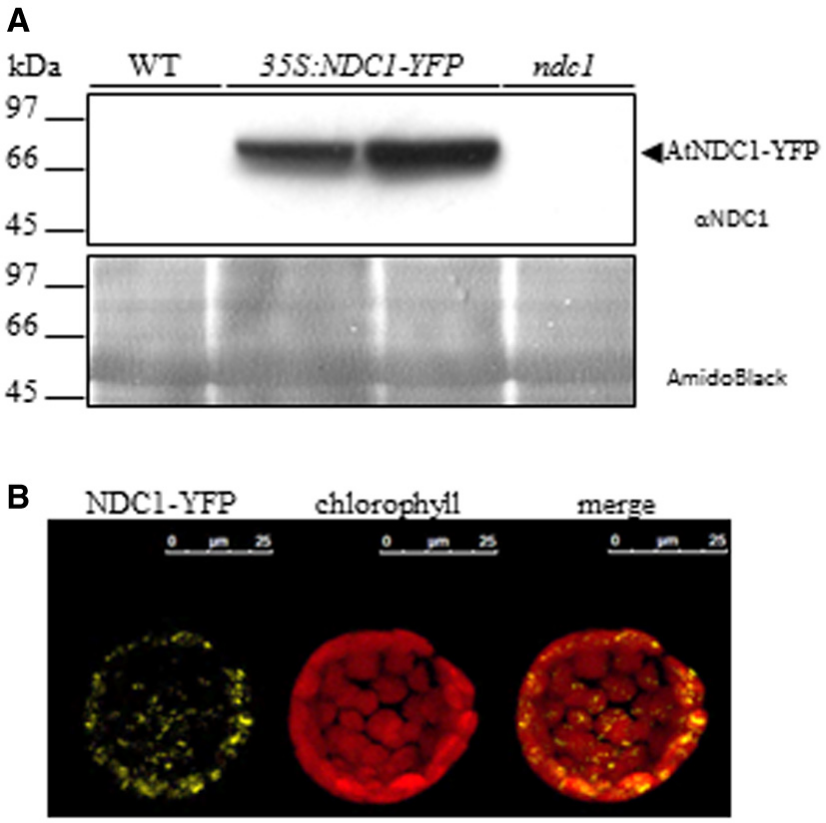
**Characterization of NDC1 overexpressing plants. (A)** Immunoblot with anti-NDC1. Twenty micrograms of total protein extract of WT, 35S:NDC1-YFP (two independent lines) and *ndc1* plants were separated by SDS-PAGE and transferred to nitrocellulose; A band corresponding to the NDC1-YFP fusion protein was detected using anti-NDC1 antibodies. The nitrocellulose membrane stained by AmidoBlack is shown to control loadings. **(B)** Protoplasts isolated from Arabidopsis overexpressing 35S:NDC1-YFP were analyzed by confocal laser microscopy (left hand panel). Autofluorescence of chlorophyll in red identifies chloroplasts (center panel). The merge of NDC1-YFP and chlorophyll fluorescence is shown in the (right hand panel).

To improve our understanding of prenylquinone biosynthesis and metabolic regulation, we used Arabidopsis wild type (WT) plants, *vte1* and *ndc1* mutants as well as 35S:VTE1-YFP and 35S:NDC1-YFP overexpressing lines exposed to either moderate light conditions or HL stress in lipidomics studies. With the exception of 35S:NDC1-YFP the different lines were characterized previously, including two independent homozygous *ndc1* mutant lines SALK_024063 and GABI_614F03 (Eugeni Piller et al., 2011) that showed no differences in phenotype or prenylquinone composition. The transgenic plants overexpressing NDC1 fused to yellow fluorescent protein (YFP) under the 35S promoter (35S:NDC1-YFP) were engineered for this study. The expression of NDC1-YFP was verified by Western blotting using an antibody against NDC1 (Figure 2A). 35S:NDC1-YFP plants but neither wild type nor *ndc1* gave a band at around 80 kDa corresponding to the predicted mass of the fusion protein. Endogenous NDC1 protein (57 kDa) was not detected in WT and overexpressing plants due to the low amount of total protein (20 μg) loaded (100 μg are necessary; Eugeni Piller et al., 2011). The 35S:NDC1-YFP plants had no apparent phenotype.

Protoplasts isolated from 35S:NDC1-YFP plants were analyzed by confocal microscopy and gave punctate fluorescence inside the chloroplasts which is in agreement with the PG localization of NDC1 (Vidi et al., 2006; Ytterberg et al., 2006; Eugeni Piller et al., 2011; Lundquist et al., 2012) (Figure 2B). Moreover, the 35S:NDC1-YFP construct was previously tested by transient expression in Arabidopsis WT protoplasts in the presence of the neutral lipid dye Nile Red which stains PG. The NDC1-YFP and Nile Red signals colocalized by confocal microscopy (Eugeni Piller et al., 2011).

### NDC1 and VTE1, two plastoglobule enzymes involved in prenylquinone metabolism

To understand the dynamics of prenylquinone synthesis under changing light conditions, we first analyzed the global lipid composition in *ndc1*, *vte1*, 35S:NDC1-YFP, 35S:VTE1-YFP, and WT genetic backgrounds.

The data obtained, using the UHPLC-QTOFMS-based method, were subjected to multivariate analysis. Using this method more than 500 different compounds were detected, not all of which could be identified (Supplementary Table 1). To investigate the difference in lipid contents, a principal component analysis (PCA) model was established from the data sets. PCA identifies and ranks major sources of variance and allows clustering of samples based on similarities and differences in the measured parameters.

**Figure 3:**
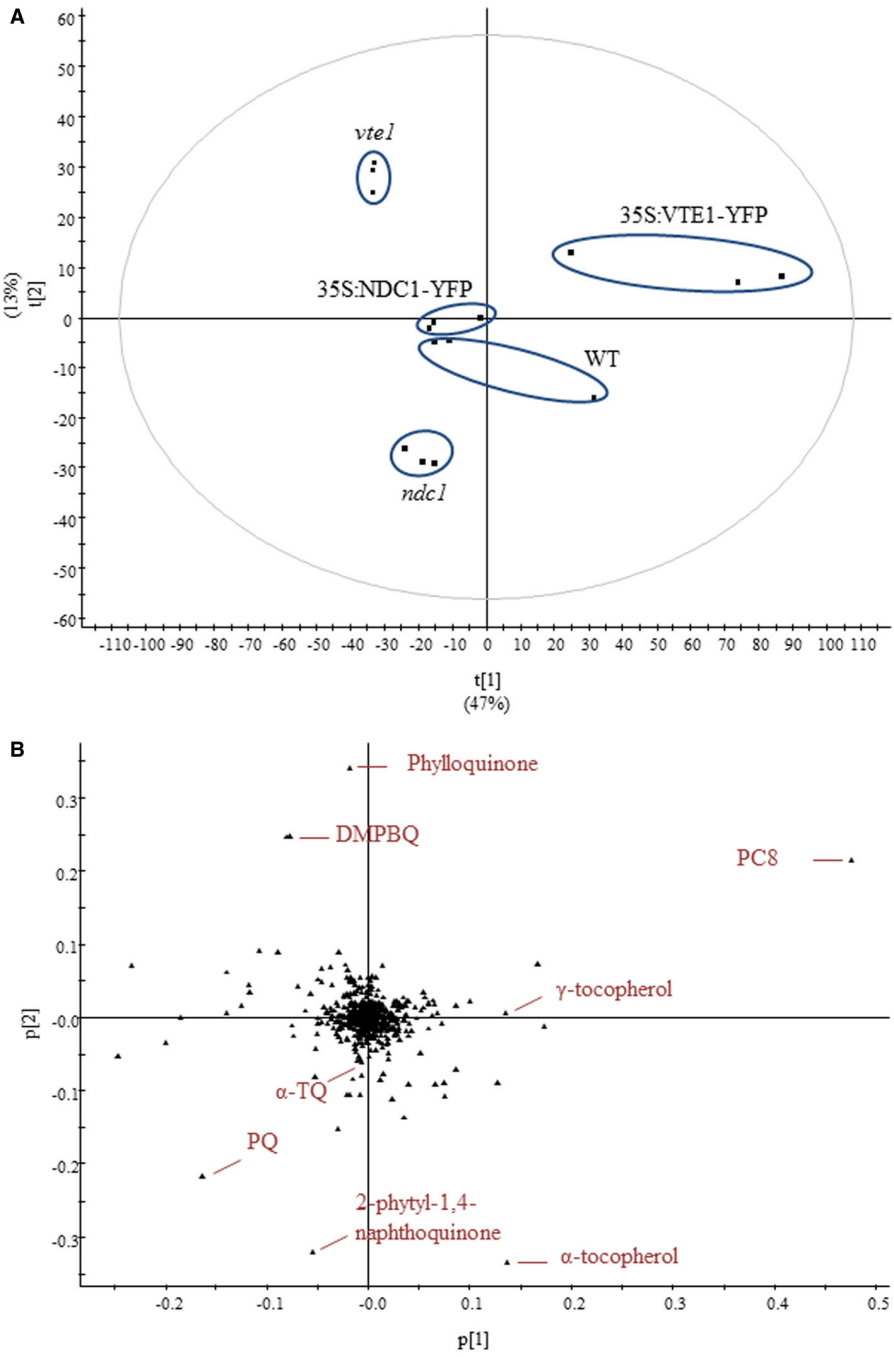
**Untargeted lipidomics showing differences in lipid profiles between five different genotypes under moderate light conditions. (A)** Principal component analysis. **(B)** Corresponding loadings plot. Lipids were extracted from WT, *ndc1*, *vte1*, 35S:NDC1-YFP and 35S:VTE1-YFP plants. Data are means of three experiments (*n* = 3).

Under moderate light conditions, PCA showed the separation of five distinct groups characteristic for each of the genotypes tested in triplicate (Figure 3A). PCA loadings revealed that prenylquinones mostly contributed to the separation of these groups (Figure 3B). Most of the other lipids extracted were near the origin, suggesting that their contribution to metabolic difference was negligible.

As expected, *vte1* plants accumulated DMPBQ lacking the chromanol ring of tocopherols. In the wild type loadings, α-tocopherol appeared instead of DMPBQ. The 35S:VTE1-YFP plants accumulated additional VTE1 products: PC8 and γ-tocopherol.

The separation of *ndc1* from the other genotypes was mainly based on the presence of the 2-phytyl-1,4-naphthoquinone, the de-methyl precursor of phylloquinone (Eugeni Piller et al., 2011). Interestingly, an accumulation of PQ was also observed in *ndc1*.

The PCA score plot indicated similar prenylquinone-lipid compositions for a representative 35S:NDC1-YFP line and the WT under moderate light conditions. This finding together with the absence of a visible phenotype suggests that the insertion of the 35S:NDC1-YFP encoding T-DNA construct was without positional effects.

**Figure 4:**
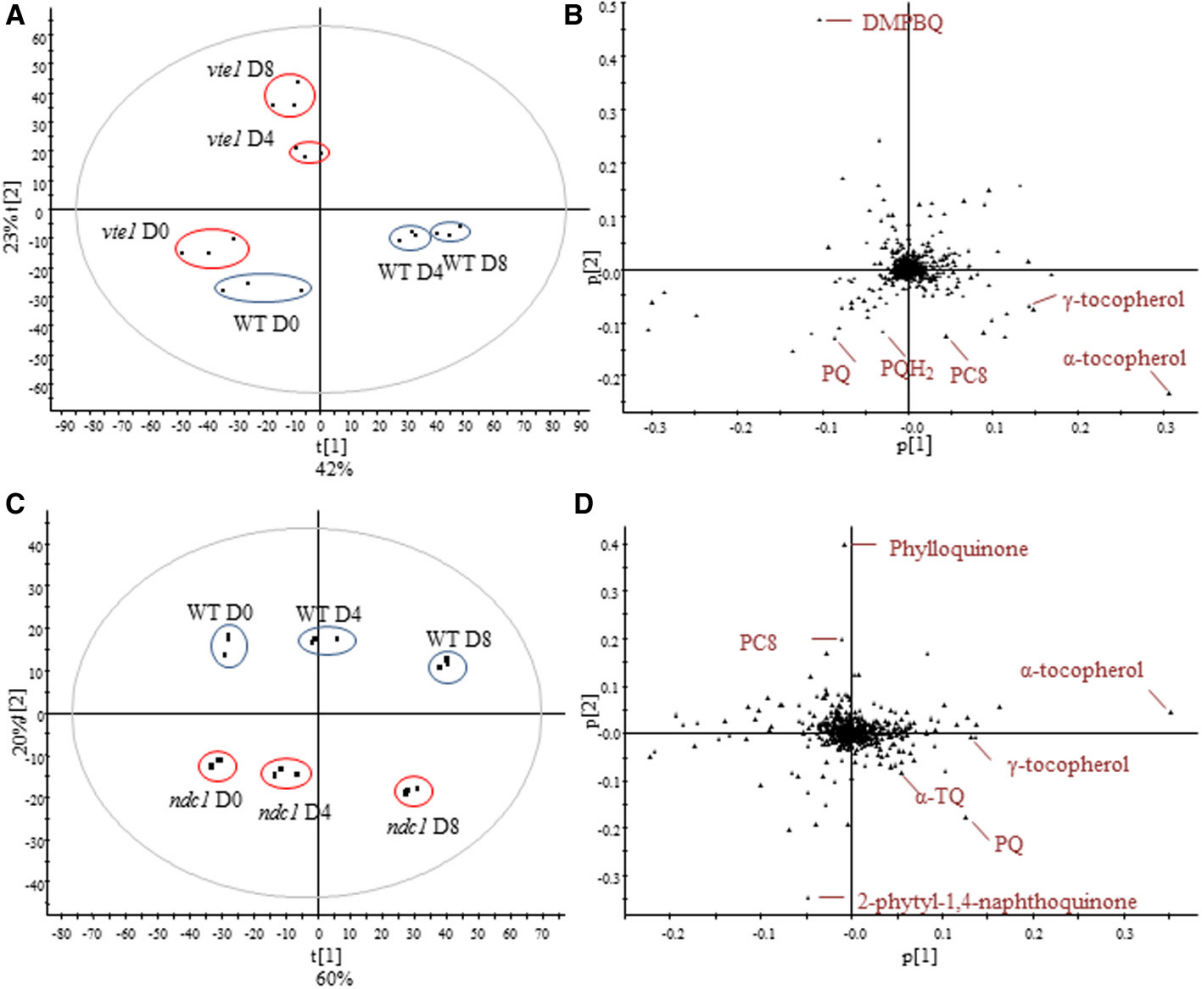
**Comparison of the lipid profile between WT and *vte1* or *ndc1* mutant plants and after HL stress. (A)** Principal component analysis showing difference between WT and *vte1* and **(B)** corresponding loadings plot. **(C)** Principal component analysis showing difference between WT and *ndc1* and **(D)** corresponding loadings plot. Plants grown under moderate light conditions (D0) were exposed to continuous HL (500 μE m^−2^ s^−1^) for 4 (D4) and 8 days (D8). Data are means of three experiments (*n* = 3).

To investigate the implication of VTE1 and NDC1 plants in prenylquinone metabolism under HL stress, we compared the lipid profiles of wild type plants with mutants after 4 and 8 days of HL exposure (Figure 4). Whereas *vte1* was blocked at the DMPBQ stage, WT plants strongly accumulated antioxidant lipids: γ-, α-tocopherol, PQ, PQH2, and PC8. (Figures 4A,B). *ndc1* differed from WT by the presence of the phylloquinone precursor and PQ but interestingly also by the accumulation of α-TQ (Figures 4C,D).

### α-tocopherol quinone, an intermediate of the tocopherol redox cycle accumulates under HL stress in *ndc1*

**Figure 5:**
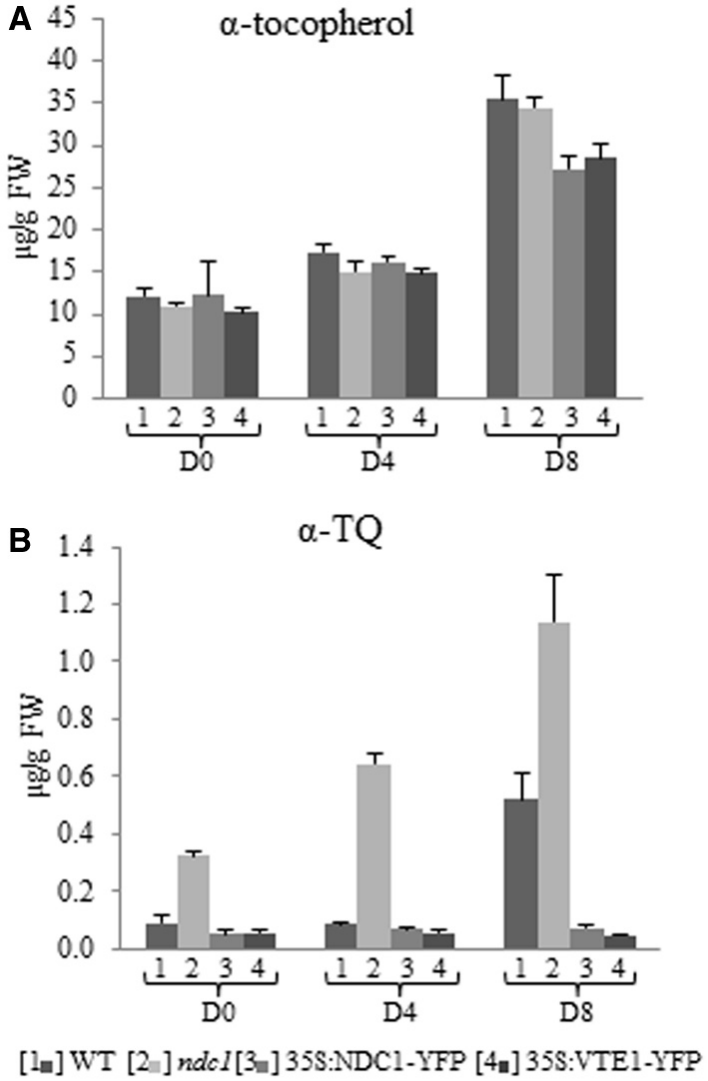
**α-tocopherol and α-tocopherol quinone quantification in leaf and after HL treatment. (A)** α-tocopherol. **(B)** α-tocopherol quinone. Lipids were extracted from [1] WT, [2] *ndc1*, [3] 35S:NDC1-YFP, and [4] 35S:VTE1-YFP plants and quantified using purified standards. Plants grown under moderate light conditions (D0) were exposed to continuous HL (500 μE m-2 s-1) for 4 (D4) and 8 days (D8). Data are means of three experiments (*n* = 3).

To assess the quantitative impact of NDC1 and VTE1 on prenylquinones after HL stress, we quantified, using pure standards, the principal compounds that were distinguished by PCA: α-tocopherol and α-TQ. As expected under HL conditions, the levels of α-tocopherol (Figure 5A) and oxidized α-TQ (Figure 5B) increased in WT, about 3 (Student’s *t*-test *p*_D0-D8_ = 0.0014) to 6-fold (*p*_D0-D8_ = 0.009) respectively after 8 days.

The level of α-tocopherol increased about 3-fold in *ndc1*, 35S:NDC1-YFP and 35S:VTE1-YFP after 8 days of HL, in the same manner as in WT (*P*_D8_WT-*ndc1* = 0.7, WT-35S:NDC1-YFP = 0.063, WT-35S:VTE1-YFP = 0.105).

With regard to the concentration of oxidized α-TQ, pronounced differences were observed between WT and mutant plants. In WT, the concentration of α-TQ increased under HL stress. However, *ndc1* accumulated at least 3 times as much α-TQ as WT under moderate and HL conditions. The finding for α-TQ was confirmed for two *ndc1* T-DNA insertion alleles (Supplementary Figure 1).

In contrast, in 35S:NDC1-YFP and 35S:VTE1-YFP the level of α-TQ remained unchanged after HL stress and was about 8 times lower than in the WT at D8.

### NDC1 is implicated in the regeneration of reduced PQ and the formation of PC8

**Figure 6:**
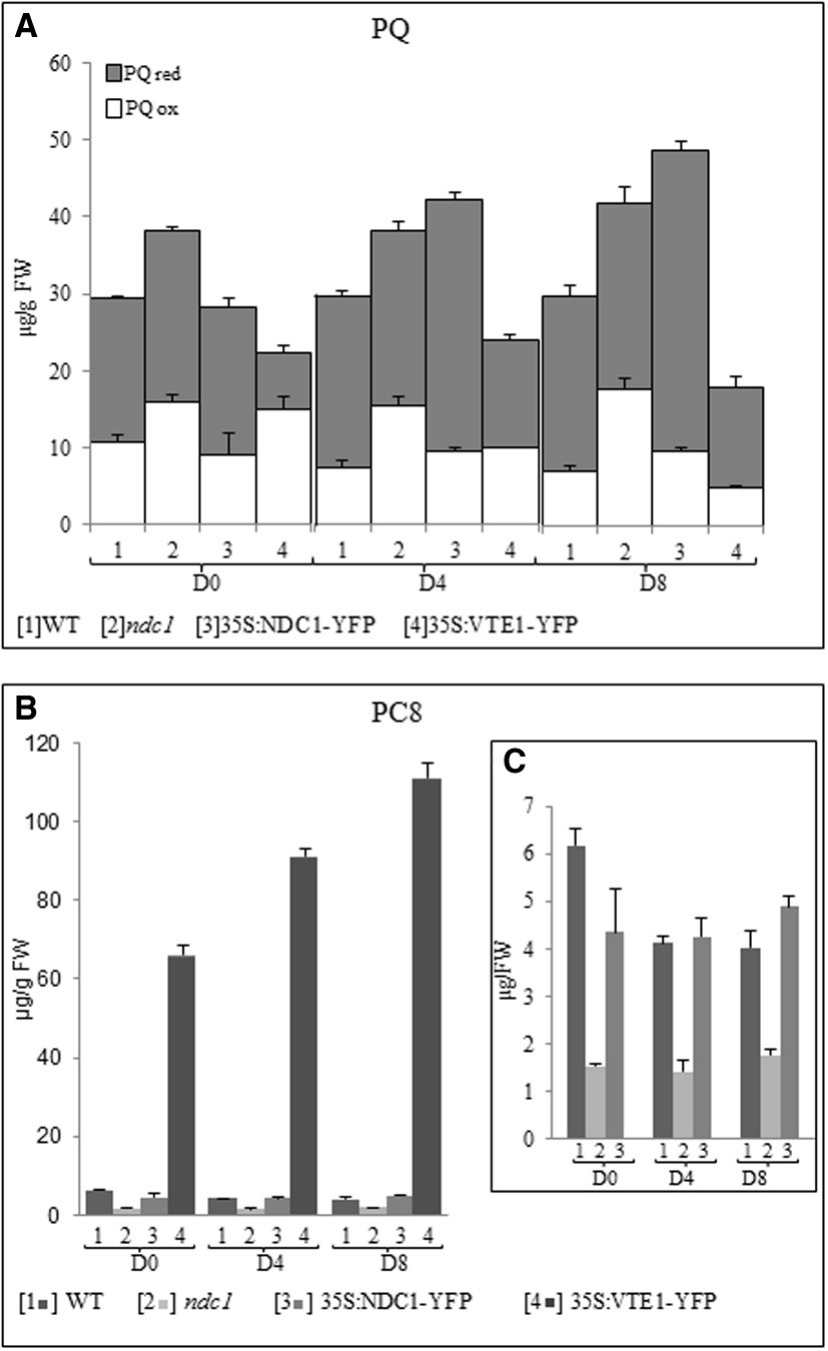
**Plastoquinone and plastochromanol quantification in leaf and after HL treatment**. Lipids were extracted from [1] WT, [2] *ndc1*, [3] 35S:NDC1-YFP, and [4] 35S:VTE1-YFP grown under moderate light conditions (D0) and after 4 (D4) and 8 (D8) days of continuous HL exposition (500μE m-2 s-1). **(A)** Total PQ was quantified using purified PQ as a standard. The white and gray bars indicating respectively the fraction of oxidized (ox) and reduced (red) PQ. **(B,C)** Quantification of PC8 using purified PC8 as a standard. Histogram presented panel **(C)** was a magnification of panels [1, 2, 3] panel **(B)**. Data are means of three experiments (*n* = 3).

The concentrations of total PQ including the proportion of the oxidized and reduced forms (Figure 6A) and of PC8 (Figures 6B,C) were measured.

In WT plants, the concentration of total PQ was unchanged after 8 days of HL (*p*_D0-D8_ = 0.471) while a slight decrease of PC8 was observed (*p*_D0-D8_ = 0.013).

In 35S:VTE1-YFP, the concentration of total PQ was generally lower than in the WT and this difference increased under HL (*p*_D0_ = 0.068, *p*_D4_ = 0.03, *p*_D8_ = 0.012). Concomitantly, PC8 concentration in 35S:VTE1-YFP was 10 times higher than WT under moderate light conditions and 27 times higher after 8 days of HL.

As expected, PC8 was not at all detectable in *vte1* (data not shown).

*ndc1* and 35S:NDC1-YFP, accumulated the highest concentrations of total PQ under HL. In *ndc1*, the concentration of oxidized PQ was significantly higher than in the WT after 8 days of HL (PQox *p*_D8_*ndc1*-WT = 0.0078). In contrast, in 35S:NDC1-YFP, the accumulation of the reduced form PQH2 made the difference (PQred *p*_D8_35S:NDC1-YFP − WT = 8.50e^−5^).

As expected, the concentration of PC8 detected in *ndc1* mutant plants was significantly lower than in the WT, but no difference was observed between WT and 35S:NDC1-YFP (*p*_D8_WT-35S:NDC1-YFP = 0.1).

### Plastoglobules, a major compartment of prenylquinone metabolism and repair

**Figure 7:**
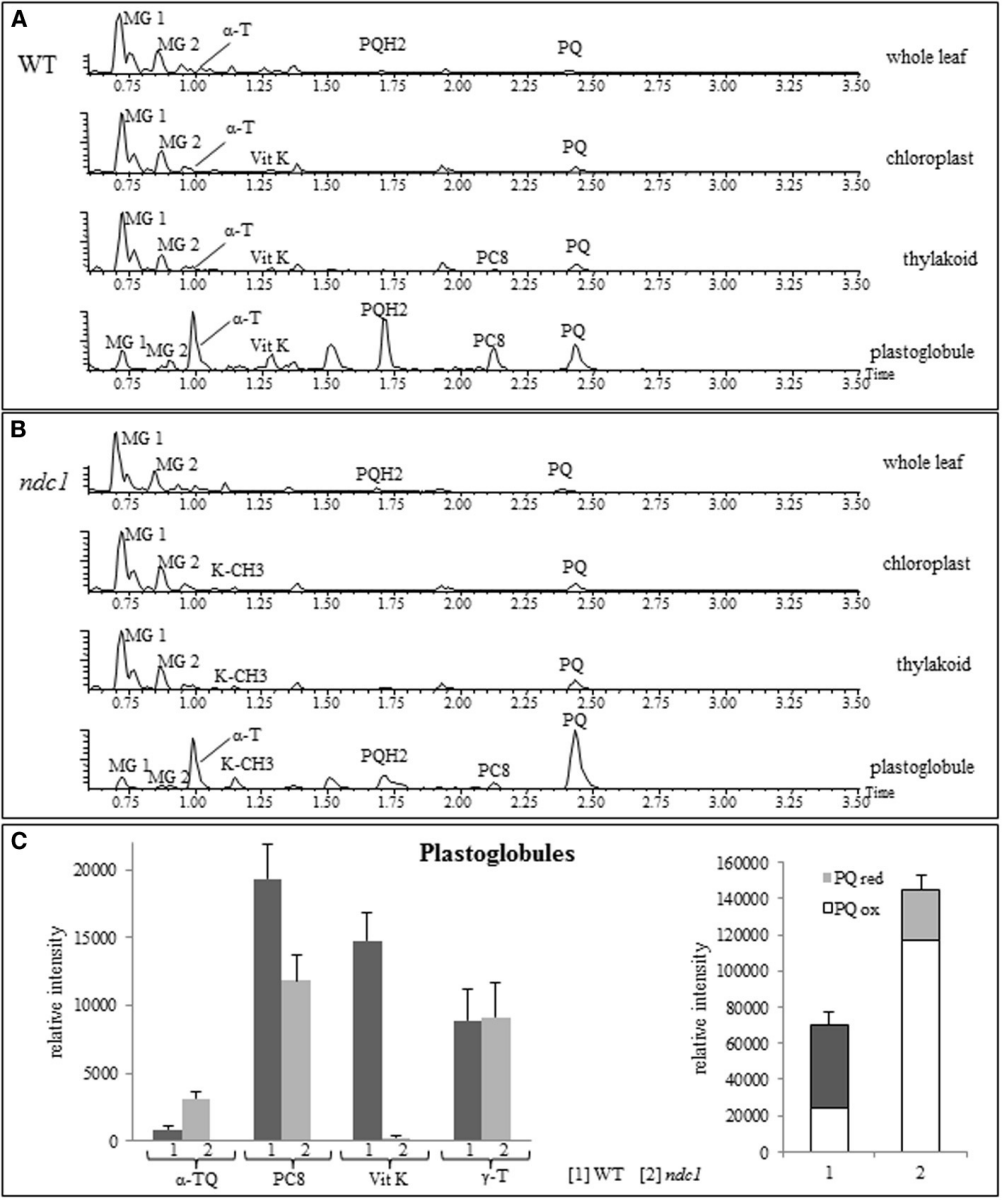
**Amount of prenylquinones in chloroplast fractions isolated from WT and *ndc1* mutant plants under HL**. UHPLC-QTOFMS chromatograms of chloroplast fractions showing prenylquinone enrichment in plastoglobules compared to other compartments **(A)** in WT and **(B)** in *ndc1* mutants. MG 1, monogalactosyldiglyceride (18:3/16:3), MG 2, monogalactosyldiglyceride (18:3/18:3), α-T (alpha-tocopherol), Vit K (phylloquinone), PQH2 (plastoquinol), PQ (plastoquinone), PC8 (plastochromanol), K-CH3 (demethylphylloquinone) after 7 days of HL. Data are means of four experiments (*n* = 4). **(C)** Quantity of α-TQ (α-tocopherol quinone), Vit K, PC8, PQ/PQH2, γ-T (gamma-tocopherol) was estimated from [1] WT and [2] *ndc1* plastoglobule fractions. Data are means of four experiments (*n* = 4).

To analyze the distribution of the prenylquinones, we isolated chloroplasts from leafs of WT and *ndc1* after 7 days of HL and separated the thylakoid membranes and the plastoglobules. We then carried out prenyllipid profiling on whole leafs, isolated chloroplast, thylakoids and plastoglobules (Figures 7A,B). The galactolipids, MG 1; monogalactosyldiglyceride (18:3/16:3) and MG 2; monogalactosyldiglyceride (18:3/18:3), abundant chloroplast membrane lipids contributing to the thylakoids as well as the lipid monolayer of plastoglobules, were used as an internal reference to assess the enrichment of prenylquinones. Clearly, the prenylquinone compounds were enriched in plastoglobules, i.e., small peaks for MG1 and −2, large peaks for α-tocopherol and PQ/PQH_2_ when compared to the thylakoid membranes in both WT and *ndc1* (approximately 30- and 40-fold, respectively). Using γ-tocopherol as an internal reference (Figure 7C), increase of α-TQ and decrease of PC8 were observed in *ndc1* PG. For α-TQ, the enrichment in PG compared to thylakoid membranes was 3- and 5-fold, respectively in WT and *ndc1*. Note that α-TQ cannot be seen as peak in the chromatograms (Figures 7A,B) due to its relatively low abundance. As expected phylloquinone (Vit K) was detectable only in WT but not in *ndc1* PG.

## Discussion

In this study we analyzed the dynamics of prenyl lipid metabolites during the change from moderate light to HL conditions using a non-targeted lipidomics approach. It is known that during acclimation to HL conditions, several prenylquinones accumulate in Arabidopsis leaves (Kobayashi and Dellapenna, 2008; Szymanska and Kruk, 2010b; Eugeni Piller et al., 2011). The four Arabidopsis genotypes used in these experiments resulted in distinct prenylquinone signatures. The most typical compounds that accumulated in each of the respective lines were: DMPBQ in *vte1*, 2-phytyl-1,4-naphthoquinone in *ndc1*, δ-, γ-tocopherol and PC8 in 35S:VTE1-YFP and finally PQH_2_ in 35S:NDC1-YFP overexpressing plants (Figure 3). Overall, α-tocopherol was the prenylquinone that increased most during the course of light stress among the 500 compounds analyzed, except in the *vte1* mutant that lacks the tocopherol cyclase (Figure 4).

This is testimony to the importance of α-tocopherol as a lipid antioxidant at the thylakoid membrane, which is subject to photooxidation and photoinhibition at PSII due to increased ROS production under HL stress (Kobayashi and Dellapenna, 2008). We previously showed that the α-tocopherol accumulation coincides with an increase in size and number of PG under HL stress (Martinis et al., 2014). This work demonstrates the implication of NDC1 in the tocopherol redox cycle. During HL stress, *ndc1* mutant plants accumulate the α-tocopherol oxidation product, α-TQ (Figure 4D). It has already been demonstrated that NDC1 is an enzyme with a wide specificity and able to reduce a range of quinolic substrates *in vitro* such as decyl-PQ, decyl-ubiquinone as well as in purified plastoglobules due to their contents of prenylquinones (Eugeni Piller et al., 2011). For α-tocopherol recycling to proceed efficiently it is likely that α-TQ must be present in the reduced form (α-TQH_2_). However, our current methodology does not allow the detection of α-TQH_2_. Nevertheless it is highly probable that NDC1 functions in the reduction of α-TQ to α-TQH_2_ to regenerate α-tocopherol. An analogous reaction mechanism has been demonstrated for the formation of γ-tocopherol, in which VTE1 closes the chromanol ring preferentially in the reduced form of DMPBQ (Grutter et al., 2006).

NDC1 is also implicated in the reduction of PQ to PQH_2_ as 35S:NDC1-YFP plants exhibited higher PQH_2_/PQ ratios (Figure 6A). Thus, NDC1 may directly influence the redox state of the PQ reservoir. Most likely, this increase in PQH_2_ concerns primarily the proportion of the plastoquinone pool present in plastoglobules that is not directly implicated in photosynthesis (Eugeni Piller et al., 2011). The observed increase of total plastoquinone in 35S:NDC1-YFP plants (Figure 6A) may be necessary to maintain sufficient oxidized PQ to allow electron transport to proceed efficiently at the thylakoid membranes.

35S:VTE1-YFP plants showed a decrease of total PQ after HL stress (Figure 6A). It is important to note that VTE1 catalyses the production of PC8 from PQH_2_ (Zbierzak et al., 2010). Therefore, the decrease of total PQ is readily explained by the pronounced accumulation of PC8 in this genotype (Figure 6B). PQH_2_ may continuously be siphoned off as a substrate of VTE1 to form PC8 explaining the decrease of PQ and the increase of PC8 in 35S:VTE1-YFP. However, in agreement with Szymanska and Kruk (2010a), our results show that the production of PC8 is not influenced by HL in WT, *ndc1* and 35S:NDC1-YFP plants but an increase is apparent in 35S:VTE1-YFP plants. Potentially, this could be explained by an increased flux of PQH_2_to PG under HL in the presence of elevated concentrations of VTE1-YFP.

In conclusion, we identify NDC1 as a novel enzyme that participates in the α-tocopherol redox cycle probably by reducing α-TQ to α-TQH_2_. In this recycling pathway, VTE1 was already known to convert TMPBQH_2_ to α-tocopherol. By hosting both NDC1 and VTE1, PG appear to play a role as metabolic repair site in the tocopherol redox cycle. This hypothesis is supported by the enrichment of α-TQ in the PG of *ndc1* compared to the WT (Figure 7C). Also, the overexpression of both NDC1-YFP and VTE1-YFP suppressed the increase of α-TQ observed in the wild type under HL (Figure 5B). This may be explained by higher levels of NDC1 or VTE1 activity that may affect the reaction kinetics of the tocopherol redox cycle and accelerate the reduction of α-TQ.

Beyond its role in tocopherol recycling and plastoquinone reduction, *ndc1* lacks phylloquinone and accumulates its de-methyl precursor instead (Figure 4D) (Eugeni Piller et al., 2011). This indicates that NDC1 has “moonlighting” role in the final methylation step of phylloquinone biosynthesis that is catalyzed by AtMENG. In the future, it will be of great interest to investigate the mechanisms of NDC1 in more detail and to determine the role of this unusual enzyme in other species.

## Author contributions

Felix Kessler, Gaétan Glauser, and Céline Besagni designed the research. Lucia Eugeni Piller, Céline Besagni, and Gaétan Glauser carried out the experimental work and statistical analyses. Felix Kessler and Céline Besagni wrote the manuscript.

### Conflict of interest statement

The authors declare that the research was conducted in the absence of any commercial or financial relationships that could be construed as a potential conflict of interest.

## Supplementary material

Supplementary Figure 1 **Amount of α-tocopherol quinone in leaf and after HL treatment**. Lipids were extracted from [1] WT and two different *ndc1* mutant lines: [2′] *ndc1* SALK_024063 and [2′] *ndc1* GABI_614F03. Plants grown under moderate light conditions (D0) were exposed to continuous HL (500 μE m-2 s-1) for 4 (D4) and 7 days (D7). Data are means of three experiments (*n* = 3).

Supplementary Table 1 **Full data set of lipid contents in Arabidopsis leaf under moderate light conditions**. Lipids were extracted by THF method from WT, *ndc1*, *vte1*, 35S:NDC1-YFP, 35S:VTE1-YFP plants and analyzed by UHPLC-QTOFMS.
