# Supplementary material for: Role of plastoglobules in metabolite repair in the tocopherol redox cycle
Source: Front Plant Sci. 2014 Jun 26;5:298. doi: 10.3389/fpls.2014.00298 (PMC4071476; doi:10.3389/fpls.2014.00298)

Supplemental Fig 1

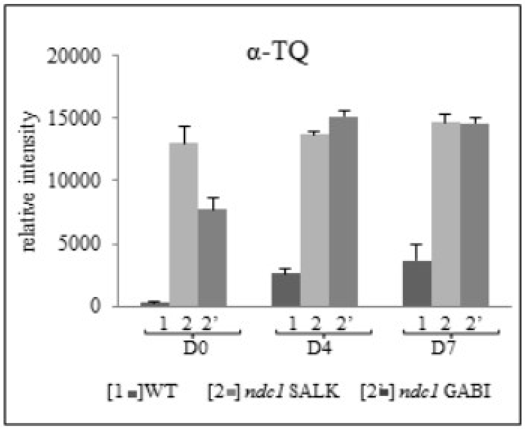

Supplement: Supplementary file 1 [file DataSheet2.DOCX]
